# YlmG1 is localized exclusively to the chloroplast envelope membrane and is involved in preprotein translocation in *Arabidopsis thaliana*

**DOI:** 10.1002/2211-5463.70222

**Published:** 2026-03-03

**Authors:** Mengyi Li, Xueyang Zhao, Masato Nakai

**Affiliations:** ^1^ Institute for Protein Research The University of Osaka Japan

**Keywords:** chloroplast, envelope membrane, protein import, TIC, translocon, YlmG

## Abstract

In green lineages, chloroplast protein import is mediated by conserved TOC and TIC translocons at the outer and inner envelope membranes, respectively. A recent structural study on TIC from *Chlamydomonas reinhardtii* revealed an additionally associated YGGT family membrane protein YlmG. Its homolog in *Arabidopsis thaliana*, YlmG1, is essential but was reported to be a thylakoid‐localized membrane protein involved in nucleoid distribution. Thus, it remains unclear whether YlmG1 participates in chloroplast protein import. Here, we show that YlmG1 is exclusively localized to the chloroplast envelope membrane where it behaves like a monomer. However, while YlmG1 is not firmly associated with TIC, it is recruited to the preprotein–translocation intermediate complex together with other TIC components, suggesting it has an important role in chloroplast protein import.

## Graphical abstract

Cytosolically synthesized chloroplast preproteins are translocated across the outer and inner envelope membranes through translocons called TOC and TIC, respectively. In green algae and plants, the TIC core is composed of essential membrane proteins, Tic12, Tic20, and Tic214. In addition, YlmG, another essential two‐transmembrane helices‐containing protein, may play a key role as a gatekeeper upon preprotein translocation through TIC.

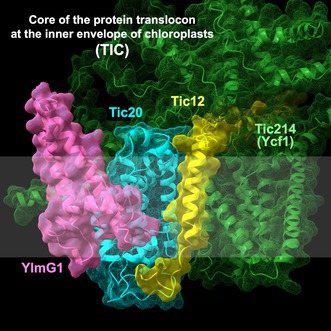

**Abbreviations**
BN‐PAGE: blue‐native polyacrylamide gel electrophoresis
CryoEM: cryogenic electron microscopy
DSP: dithiobis(succinimidyl propionate)
LC–MS/MS: liquid chromatography–tandem mass spectrometry
TIC: translocon at the inner envelope membrane of chloroplast
TOC: translocon at the outer envelope membrane of chloroplast

Most chloroplast proteins are nucleus‐encoded, synthesized in the cytosol as a larger precursor protein called preprotein and are translocated across the outer and inner envelope membranes successively through TOC and TIC translocons, respectively [1, 2, 3], with the aid of the inner envelope membrane‐localized ATP‐dependent pulling motor, the Ycf2/FtsHi complex [4]. TOC and TIC constituents have been extensively characterized by means of biochemistry, cell biology, and molecular genetics as well as structural biology. Whereas extant Rhodophyta and Glaucophyta, early lineages of chloroplast‐containing eukaryotic algae, likely retain evolutionary primitive types of these translocons [5], green lineages, including Chlorophyta and Embryophyta, plants, possess well conserved TOC and TIC constituents as well as Ycf2/FtsHi motor complex constituents [4, 6, 7, 8, 9]. TOC consists of a central integral membrane protein component Toc75 and two GTP‐binding proteins Toc159 and Toc33, among which Toc75 and Toc159 form a mixed beta‐barrel channel in the outer envelope membrane and Toc159 and Toc33 function as receptors for preproteins [1, 2, 3]. Central TIC constituents had long been a matter of debate [10, 11, 12, 13, 14]; however, recent extensive biochemical and structural studies have underscored the central role of Tic20 in the formation of TIC core [4, 6, 7, 9]. Both CryoEM structures of TIC from *Chlamydomonas reinhardtii* [15, 16] and from *Arabidopsis thaliana* [9] revealed that Tic20 bears four transmembrane helices and forms an inner envelope membrane‐embedded part of TIC with other two essential components, namely Tic12 [17] and Tic214 (Ycf1), which contain one and six transmembrane helices, respectively. Tic214 forms a large intermembrane space domain of TIC, which is intertwined with two additional essential proteins, Tic56 and Tic100. While Chlamydomonas TIC and Arabidopsis TIC show overall structural similarities, there are several remarkable differences [9, 15, 16]: (i) *Chlamydomonas* TIC forms a stable supercomplex with TOC in which a small part of TIC214 is integrated into the Toc75/Toc159 mixed barrel, whereas *Arabidopsis* TIC does not; (ii) *Chlamydomonas* TIC contains another essential two‐transmembrane helices‐containing YlmG which binds to a transmembrane segment of Tic20 located opposite to the Tic214/Tic12‐interacting face of Tic20 [8]. Among four YlmG homologous proteins found in *Arabidopsis*, YlmG1‐1 (At3g07430; hereafter referred to as YlmG1), which is the closest homolog of *Chlamydomonas* YlmG, was shown to be an indispensable protein required for chloroplast biogenesis [18]. On the contrary, another study demonstrated that YlmG1 was exclusively localized on the thylakoid membrane and was involved in nucleoid distribution [19]. Thus far, involvement of YlmG1 in chloroplast protein import in plants has not been investigated. In this report, we revisited suborganellar location of YlmG1 in *Arabidopsis* chloroplasts and analyzed its molecular interaction with TIC as well as with translocating preproteins.

## Materials and methods

### Plant growth and isolation of chloroplasts

*Arabidopsis thaliana* (*Col‐*0) was used as the wild‐type. The *PA2‐TIC20* line expressing Protein A‐tagged Tic20‐I fusion protein in the homozygous *tic20‐I* knock‐out mutant background was also used [6]. *Arabidopsis* plants were grown on solid media containing Murashige and Skoog salts (Sigma‐Aldrich, St. Louis, MO, USA), Gamborg B5 vitamin (Sigma‐Aldrich), 2% (w/v) sucrose, pH 5.7, and 0.3% (w/v) phytagel (Sigma‐Aldrich) for 21–26 days under a cycle of 16 h light (light intensity of 100–150 μmol m^−2^ s^−1^) at 23 °C and 8 h of darkness at 21 °C. Isolation of intact chloroplasts was performed as described previously [17]. To obtain suborganellar fractions, isolated chloroplasts were lysed in a hypotonic buffer (10 mm HEPES‐KOH, pH 8.0, and 4 mm MgCl_2_). After centrifugation at 3000 ***g*** for 5 min to obtain thylakoids as a pellet, the supernatant was layered over a sucrose step gradient composed of 1 m and 0.46 m sucrose (in 10 mm Tricine‐KOH, pH 7.5, and 2 mm EDTA) and was centrifuged at 70 000 ***g*** for 1 h. The envelope fraction was collected from the boundary between the two layers, and the stromal fraction was recovered from the solution remained at the top of the gradient. Purification of PA2‐TIC complex was performed using chloroplasts isolated from the PA2‐TIC20 transgenic line as described previously [17]. Gel filtration, SDS/PAGE, Blue‐Native PAGE, and immunoblotting were carried out with conventional methods [17, 20]. For immunodetection of YlmG1, electrotransfer of proteins after gel electrophoresis was performed using CAPS buffer with nitrocellulose membrane (0.2 μm; Bio‐Rad, Hercules, CA, USA). Furthermore, to activate and enhance the antigenicity on the membrane, in some cases, the transferred membranes were heat‐treated in hot water for several minutes before blocking.

### *In vitro* import of preproteins and purification of translocation intermediate complexes

Purification of model preproteins pFd‐TEV‐ProteinA and pSSC‐HAPA was as described previously [6, 17]. For *in vitro* import experiments, isolated intact *Arabidopsis* chloroplasts were incubated with purified preproteins in the presence of 0–3 mm ATP in buffer containing 50 mm HEPES‐KOH, pH 8.0, and 330 mm sorbitol for 20 min at 25 °C. To purify translocation intermediate complexes, after several washings, chloroplasts were solubilized with 1% digitonin buffer containing 150 mm NaCl, 50 mm Tris–HCl, pH 7.5, and 5% [v/v] protease inhibitor cocktail (PIC) (Roche) for 2 h with rotation at 4 °C. Insoluble materials were removed by centrifugation at 21 500 ***g*** for 30 min at 4 °C. Purification of the translocation intermediate complexes was performed using anti‐PA‐tag Sepharose (Fujifilm Wako, Osaka, Japan) or IgG‐Sepharose (Sigma‐Aldrich) according to the published methods [4, 17]. The subsequent LC–MS/MS analysis was described previously [4].

### Preparation of antibodies

The coding sequence corresponding to amino acid residues 73–232 of YlmG1 (At3g07430) was PCR‐amplified and cloned into the pGEMEX‐1 vector (Promega, Madison, WI, USA) with a short DNA segment for the addition of a hexa‐histidine tag. The recombinant Gene10‐YlmG1 fusion protein was purified under urea‐denaturing conditions using His‐Bind Resin (Novagen, Madison, WI, USA) and was used as an antigen for immunization with rabbits to obtain anti‐YlmG antibody (α846). To obtain anti‐*Oryza sativa* YlmG1 (Os07g08770) antibody (α19‐2), the coding sequence corresponding to amino acid residues 70–175 was cloned into the pCold‐I vector (Takara, Shiga, Japan) and was used as an antigen as described above. A synthetic peptide corresponding to amino acid residues 221–232 of Arabidopsis YlmG1 was also used as an antigen after conjugation to carrier protein (Keyhole Limpet Hemocyanin (KLH)) to obtain another anti‐YlmG1 antibody (α3411). The specific anti‐YlmG1 antibodies were further purified using polyvinylidene fluoride (PVDF) membranes blotted with the GST‐YlmG1 fusion protein containing amino acid residues 73–232 of YlmG1, which was expressed in and purified from *Escherichia coli* cells using pCold‐GST vector (TAKARA). Other antisera used in this study were described in previous studies [4, 17]. The commercially available monoclonal antibody against the HA tag (clone HA‐7; Sigma‐Aldrich; clone 16B12; BioLegend, San Diego, CA, USA) was also used.

## Results

### YlmG1 is exclusively localized in the envelope membrane of chloroplast in *Arabidopsis*

**Fig 1:**
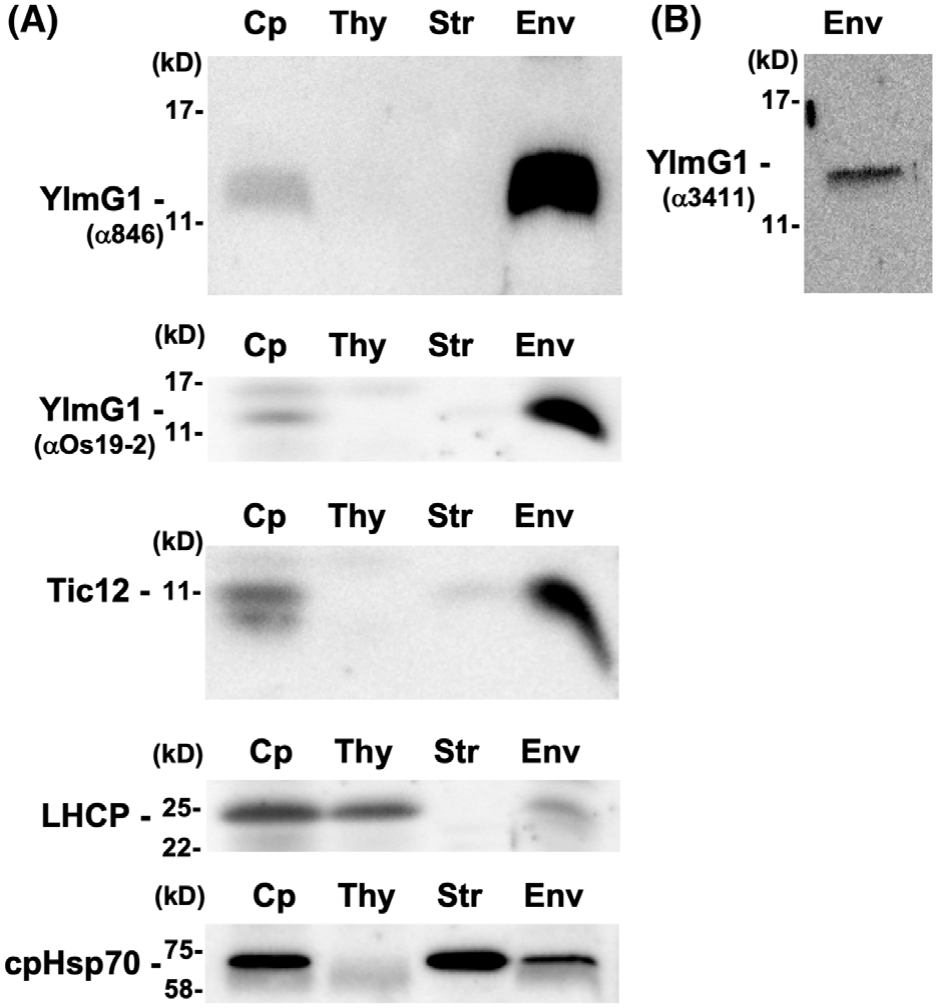
YlmG1 protein encoded by At3g07430 localizes to the chloroplast envelope membrane in *Arabidopsis*. (A) Envelope localization of YlmG1. Intact chloroplasts (Cp) isolated from *Arabidopsis* were fractionated into thylakoids (Thy), stroma (Str), and envelope (Env). Fractions were analyzed by SDS/PAGE followed by immunoblotting using specific antisera for respective proteins. LHCP (chlorophyll a/b‐binding protein, cpHsp70 (chaperone), and Tic12 were analyzed for representative thylakoid, stromal, and envelope proteins, respectively. Two different antibodies, α846 and αOs19‐2 were used to detect YlmG1. (B) Detection of envelope YlmG1 using another antibody (α3411).

In the earlier study, anti‐YlmG1 antibody (raised against internal peptides) was used to determine the suborganellar location of YlmG1 in *Arabidopsis* chloroplasts by means of immunoblotting and immunofluorescent microscopy [19]. Since this antibody was no longer available, we tried several different peptides as well as recombinant proteins derived from YlmG1 protein sequence used as antigens to raise specific antibodies. However, it was rather difficult to obtain an antibody that was strong enough to be used for standard biochemical analyses, such as immunoblotting, probably because YlmG1 is a small hydrophobic protein. Only a few antisera that we tried contained specific antibodies, but even so, their antibody titers were not high, so we needed to concentrate the specific antibodies by affinity purification using antigen‐bound matrices. One such antibody, anti‐YlmG1 (referred to as α846) raised against amino acid residues 73–232 of *Arabidopsis* YlmG1, recognized an approximately 13–15 kDa protein in isolated *Arabidopsis* chloroplasts upon SDS/PAGE followed by immunoblotting (Fig. 1A). This protein was found exclusively in the envelope fraction upon suborganellar fractionation of chloroplasts. The same envelope‐localized protein band of 13–15 kDa was commonly detected by another anti‐YlmG1 antibody raised against amino acid residues 70–175 of homologous YlmG1 (Os07g08770) from *Oryza sativa* (referred to as αOs19‐2) (Fig. 1A) as well as by that raised against a synthetic peptide corresponding to the C‐terminal amino acid residues 221–232 of *Arabidopsis* YlmG1 (referred to as α3411) (Fig. 1B). Therefore, despite the fact that the previous study reported its thylakoid localization, we conclude that YlmG1 is envelope‐localized in Arabidopsis.

### YlmG1 is not firmly attached to the TIC complex

**Fig 2:**
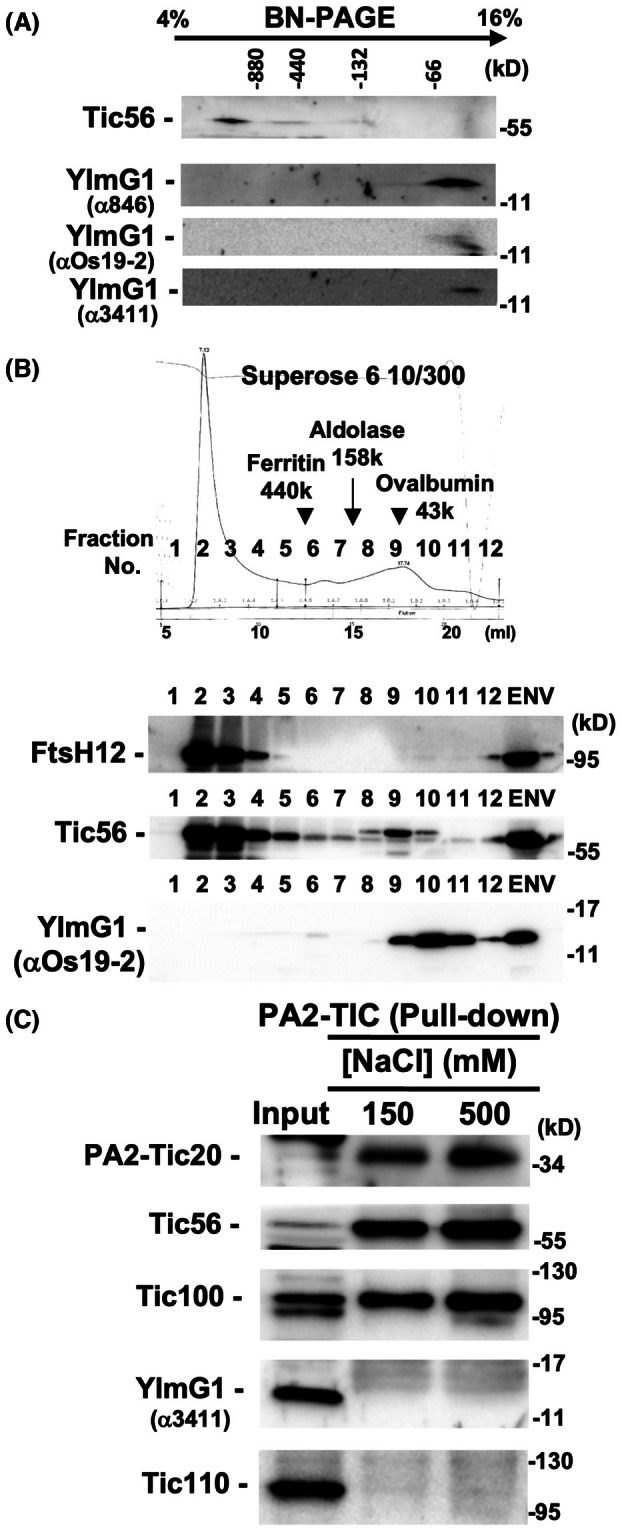
YlmG1 exists as most likely a monomer apart from TIC complex. (A) 2D‐BN/SDS/PAGE analysis of YlmG1. Intact *Arabidopsis* chloroplasts were solubilized using 1% (w/v) digitonin‐containing buffer and were applied to 4–16% BN‐PAGE as the first dimension followed by SDS/PAGE for the second dimension. Immunoblotting was performed to detect each protein. (B) Size‐exclusion chromatography of solubilized envelope membrane proteins. Chloroplasts were solubilized as in (A) and were applied on Superose 6 (10/300; Cytiva) size‐exclusion column. Eluted fractions were analyzed by immunoblotting. The chromatographic profile monitored by absorbance at 280 nm and eluted positions of molecular size markers are shown at the top. (C) Pull‐down analysis to assess the presence of YlmG1 in the TIC complex. Chloroplasts isolated from the transgenic *Arabidopsis* line expressing the Protein A‐tagged Tic20 (PA2‐TIC) were solubilized as above and the TIC complex was purified using IgG‐agarose. Binding and elution were conducted in the presence of 150 or 500 mm NaCl as indicated. Eluted proteins and input sample were analyzed by immunoblotting.

To analyze the molecular status of YlmG1 protein in the envelope membrane, first, we performed 2D‐BN‐PAGE/SDS/PAGE followed by immunoblotting analysis after solubilization of the chloroplast envelope membrane by a mild detergent, digitonin. Tic56, one of the core components of TIC, migrated around 1 MDa on the BN‐PAGE gel as reported previously (Fig. 2A) [4, 6]. By contrast, the YlmG1 protein band detected by anti‐YlmG1 (α846) appeared near the front of the BN‐PAGE gel (< 60 kDa). Similar migration of YlmG1 was observed when using anti‐YlmG1 (αOs19‐2) or (Os3411) (Fig. 2A). BN‐PAGE is the gel‐electrophoretic method to separate solubilized membrane protein complexes by size under nondenaturing conditions. However, the presence of Coomassie Brilliant Blue dye (G‐250) during BN‐PAGE, which binds to certain amino acid residues in proteins, may affect the integrity of assembly of solubilized membrane protein complexes. Thus, next we performed size‐exclusion chromatography using a Superose 6 column to separate the envelope membrane protein complexes solubilized by digitonin (Fig. 2B). Under the condition, we used, Tic56 eluted mostly in the void fraction where FtsH12, one of the Ycf2/FtsHi motor complex components, was also found eluted, suggesting that TIC and the Ycf2/FtsHi motor might form certain larger supercomplexes as reported previously. YlmG1 was eluted in clearly distinct fractions near the column volume corresponding to fractions of small protein molecules (< 43 kDa), indicating that YlmG1 exists as a distinct entity from TIC.

To confirm this further, we performed a pull‐down experiment using digitonin‐solubilized chloroplast envelope membrane fraction prepared from the transgenic *Arabidopsis* line expressing the Protein A‐tagged form of Tic20 (Fig. 2C). Tic56 and Tic100 were co‐purified with the Protein A‐tagged Tic20 even under the presence of high salts (500 mm NaCl), whereas Tic110, which is a well‐known abundant inner envelope marker protein, was not, as previously demonstrated [6]. YlmG1 was not co‐purified with TIC even under the physiological salt condition (150 mm NaCl), confirming that YlmG1 is not a stably associated constituent of TIC.

### YlmG1 is recruited to the translocation intermediate complex together with other TIC components

**Fig 3:**
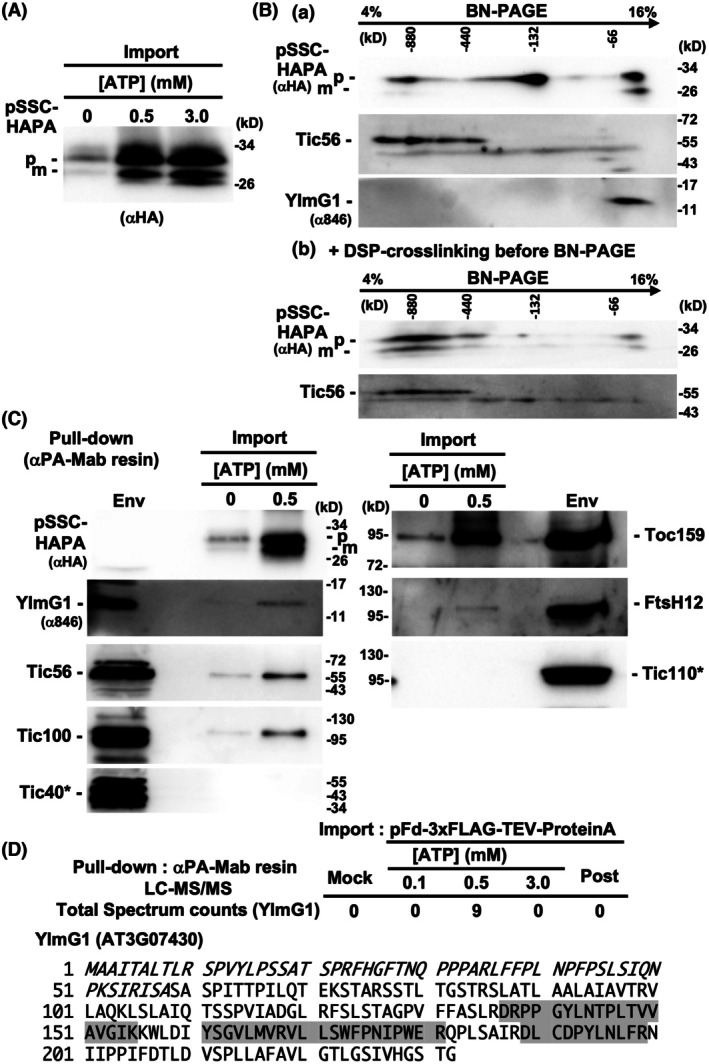
YlmG1 associates with translocating preproteins with other TIC constituents. (A) *In vitro* import of model preprotein, pSSC‐HAPA, into isolated intact chloroplasts from *Arabidopsis*. Import reactions were conducted in the absence or in the presence of ATP (0.5 or 3.0 mm) and analyzed by immunoblotting with anti‐HA tag antibody (αHA) to detect pSSC‐HAPA: p, precursor form; m, imported processed mature form. (B) 2D‐BN/SDS/PAGE analysis of imported preproteins. (A) After import reactions in the presence of 0.5 mm ATP, washed chloroplasts were solubilized by 1% digitonin and analyzed by 2D‐BN/SDS/PAGE followed by immunoblotting. (B) As in (A), but before solubilization, chloroplasts were treated with 0.1 mm of crosslinker DSP (dithiobis(succinimidyl propionate)) for 1 h on ice followed by quenching with Tris base. Note that DSP is a cleavable crosslinker so that the crosslinked adducts are stable during BN‐PAGE but are reductively cleaved before SDS/PAGE by incubating the BN‐PAGE gel in the presence of excess reductant, 2‐mercaptoethanol. YlmG was unable to be detected by immunoblotting after DSP crosslinking, probably because certain modifications of amino acid side‐chains in YlmG by DSP might affect its antigenicity even after cleavage. (C) Pull‐down experiments with pSSC‐HAPA after *in vitro* import into *Arabidopsis* chloroplasts in the absence or the presence of ATP (0.5 mm) as in Fig. 2C, but using anti‐PA tag monoclonal antibody‐conjugated resin (αPA‐Mab resin) under the presence of 150 mm NaCl. (D) Identification of YlmG as the translocation intermediate‐associating protein by LC–MS/MS. The model preprotein pFd‐3xFLAG‐TEV‐Protein A was used for *in vitro* import experiments with *Arabidopsis* chloroplasts in the presence of 0.1, 0.5, or 3.0 mm ATP, and their translocation intermediates were purified using IgG‐agarose and analyzed by LC–MS/MS [4]. Mock‐purified sample (without the addition of preproteins) and another negative control sample denoted as Post (addition of excess preproteins just prior to purification but after solubilization) were also analyzed (see ref. [4] for details). Sequence coverage of YlmG by the LC–MS/MS analysis is shown at the bottom. MS‐identified peptides are shown with gray backgrounds and the predicted transit sequence is shown in italics.

Finally, we examined whether YlmG1 would interact with an incoming preprotein during preprotein translocation across the inner envelope membrane. To this end, we performed *in vitro* import experiments using isolated intact chloroplasts prepared from *Arabidopsis* with the purified model preprotein pSSC‐HAPA containing a transit peptide derived from pRbcS, a rubisco small subunit precursor, and the tandem HA and PA tags (Fig. 3A). Depending on the increasing concentration of ATP in the import reactions, the amount of imported processed form of pSSC‐HAPA (mSSC‐HAPA) was increased, confirming its ATP‐dependent translocation across the envelope membranes. Under the presence of the moderate concentration of ATP (0.5 mm) during import, after solubilization with digitonin, a fraction of pSSC‐HAPA was found in larger complexes of around 1 MDa or even larger upon BN‐PAGE separation where Tic56 was also migrated (Fig. 3B, (a)). YlmG1 protein remained found near the front of the BN‐PAGE gel. However, this might be simply due to the fairly low amount of translocation intermediates formed by *in vitro* import experiments compared with the abundance of endogenous proteins in the envelope membrane. DSP (dithiobis(succinimidyl propionate))‐crosslinking of solubilized membrane protein complexes before BN‐PAGE led to significant increase of pSSC‐HAPA appeared around the 1 MDa area (Fig. 3B, (b)), indicating the formation and significant accumulation of translocation intermediates complexes under this import condition we used. Thus, we next performed pull‐down experiments using anti‐PA‐tag affinity agarose matrices after solubilization of translocation intermediate complexes by the mild detergent digitonin but without DSP crosslinking (Fig. 3C). Tic56 and Tic100 were pull‐down by this method depending on the amounts of translocation intermediate complexes formed under the different ATP concentrations during import. YlmG1 was similarly co‐purified with these other TIC components. By contrast, neither Tic110 nor Tic40, not a constituent of Tic20‐centered TIC, was pull‐down under this condition, indicating that the observed co‐purification of YlmG1 in the translocation intermediate complexes can be considered as specific.

We previously purified translocation intermediates after *in vitro* import of another model preprotein, pFd‐TEV‐ProteinA carrying a transit peptide of ferredoxin, into isolated *Arabidopsis* chloroplasts and analyzed extensively by LC–MS/MS to identify the associated components [4]. This method was very effective to detect all of the TOC, TIC, and Ycf2/FtsHi motor complex components. However, for relatively smaller hydrophobic proteins, even if they play an important physiological function, they are generally difficult to detect by MS and to be distinguished from nonspecific binders of high abundance solely based on MS spectra counts, so that detailed biochemical analyses must be carried out. From this point of view, our previous biochemical investigations on a relatively small protein picked up from the MS data led to the successful identification of Tic12 [17]. Similarly, we had noticed the presence of YlmG1 MS data in the list of MS‐identified translocation intermediate‐associating proteins and thus had already started the biochemical analyses on YlmG1 before the CryoEM structure of TIC from *Chlamydomonas* appeared in public. As a matter of fact, as shown in Fig. 3D, YlmG1 protein was detected by the MS [4] as one of the translocation intermediate‐associating proteins only when a moderate level of ATP (0.5 mm) had been included during *in vitro* import reactions, under which translocation intermediates accumulated at the inner envelope membrane. Although its MS‐identified peptide spectra counts were not that high, combined with the additional biochemical data shown in this study (Fig. 3C), these MS data also support our conclusion that YlmG1 participates in the formation of translocation intermediate complexes upon preprotein translocation across the chloroplast envelope membrane.

## Discussion

Taken together, we conclude that, in *Arabidopsis*, YlmG1 protein is exclusively localized in the envelope membrane of chloroplast. While YlmG1 is not a stably associated component of TIC and exists most likely as a monomeric form, it is recruited to the preprotein–translocation intermediate complexes together with other TIC components. Since YlmG1 was shown to be absolutely required for chloroplast biogenesis as well as for plant viability [18], this protein is assumed to play an important and indispensable role in preprotein translocation across the inner envelope membrane of chloroplast. This is a clear discrepancy with the previous report that YlmG1 is exclusively localized on the thylakoid membrane and plays a role in nucleoid distribution [19]. The reason for the latter can be explained if the knock‐down of *YLMG1* in Arabidopsis affects the import efficiency of certain proteins involved in nucleoid distribution. A possible reason for the former might be related to a considerable difficulty we experienced in obtaining a strong enough antibody that can specifically recognize the endogenous YlmG1 protein. To overcome this, in our study, we tried and selected several antisera of different sources, which commonly recognized the same envelope‐localized protein, confirming the exclusive localization of YlmG1 in the chloroplast envelope membrane with confidence.

**Fig 4:**
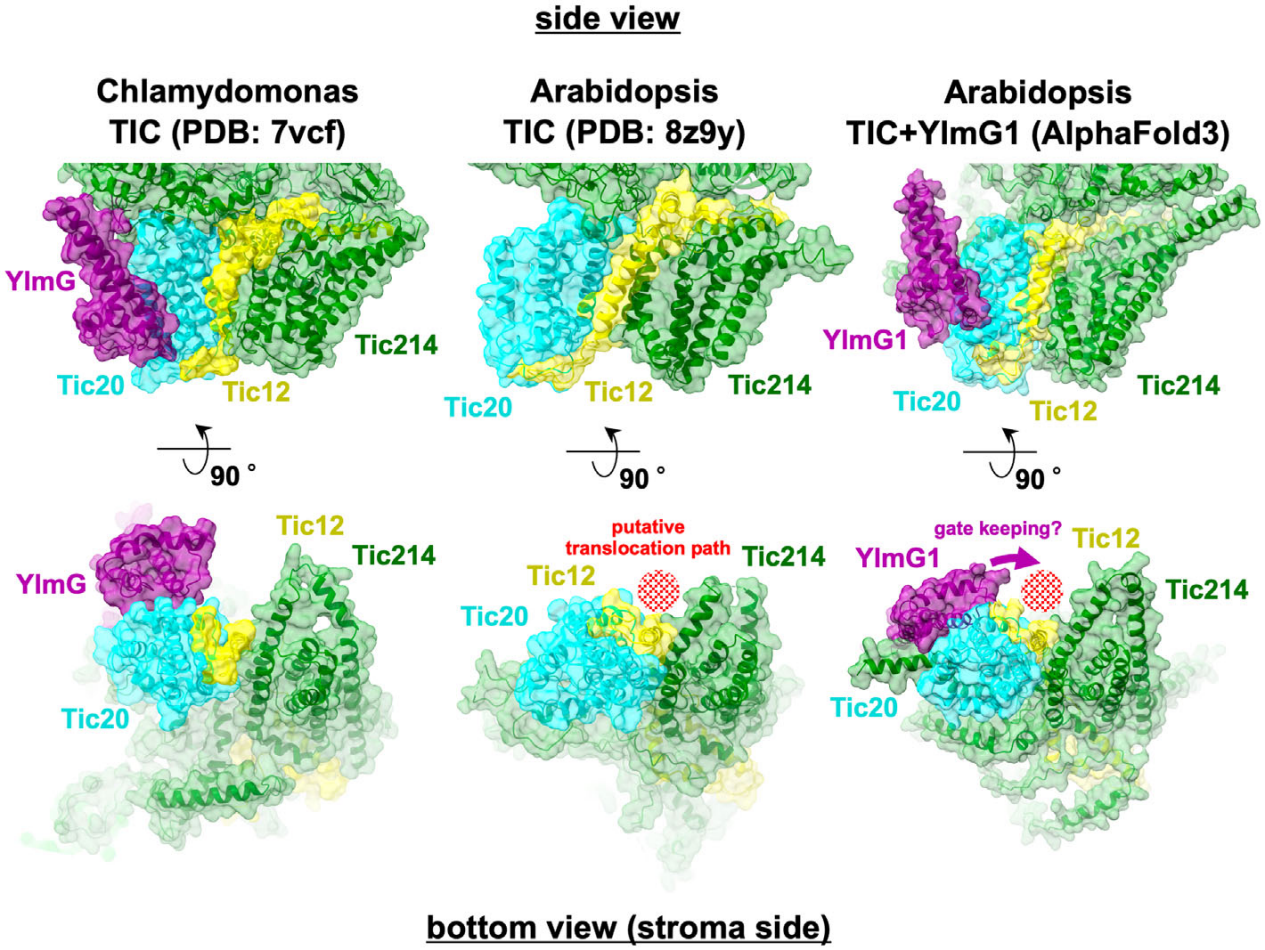
Model for the functional participation of YlmG during preprotein translocation through TIC. Structural comparison between the inner envelope membrane‐embedded parts of TIC complexes from *Chlamydomonas* (PDB: 7vcf, left) and *Arabidopsis* (PDB: 8z9y, center). For simplicity, only Tic12 (yellow), Tic20 (cyan), Tic214 (green), and YlmG (magenta) are shown and their inner envelope embedded parts are focused and shown from side view and from bottom view (from the stroma). A putative preprotein translocation path through the concave cavity formed by Tic20‐Tic12‐Tic214 transmembrane segments is indicated in red. The AlphaFold 3‐predicted structure of Tic12, Tic20, and Tic214 together with YlmG1 (amino acid residues 127–232) is shown similarly (right). The predicted model was generated using the AlphaFold server (https://alphafoldserver.com/) powered by AlphaFold3 [21]. During translocation, YlmG1 associates with TIC and possibly acts as a gatekeeper to avoid and/or enhance the lateral release of incoming proteins to the lipid phase of the inner envelope membrane.

On the contrary, our observations are in good agreement with the recent finding of the CryoEM structure of TIC complex from *Chlamydomonas reinhardtii* that YlmG is found associated with Tic20, the central key protein of TIC (Fig. 4, left) [15, 16]. Tic20, a four transmembrane helices‐containing protein, forms a membrane core of TIC with Tic214 (Ycf1), a six transmembrane helices‐containing protein. Tic12 (also known as Simp1), a single transmembrane helix containing protein of essential function, is stuck in between Tic20 and Tic214 (Fig. 4, left). Tic20, Tic12, and Tic214 form a half‐opened concave cavity in the inner envelope membrane. The similar concave cavity can be found in the recently determined CryoEM structure of TIC from *Arabidopsis* which lacks bound YlmG1 (Fig. 4, center) [9]. Our previous *in vitro* protein import study revealed that both Tic20 and Tic12 were crosslinked with the transit peptide moiety of translocating pSSC‐HAPA [17], suggesting that preproteins may traverse the inner envelope membrane in close proximity with the above‐mentioned concave cavity formed by Tic20, Tic12, and Tic214 (Fig. 4, center). In the *Chlamydomonas* TIC structure, YlmG binds to Tic20 at the rim of the concave cavity, and because of this experimentally determined structure, the AlphaFold 3‐predicted structure of *Arabidopsis* TIC and YlmG1 also exhibit a similar arrangement (Fig. 4, right) [21].

**Fig 5:**
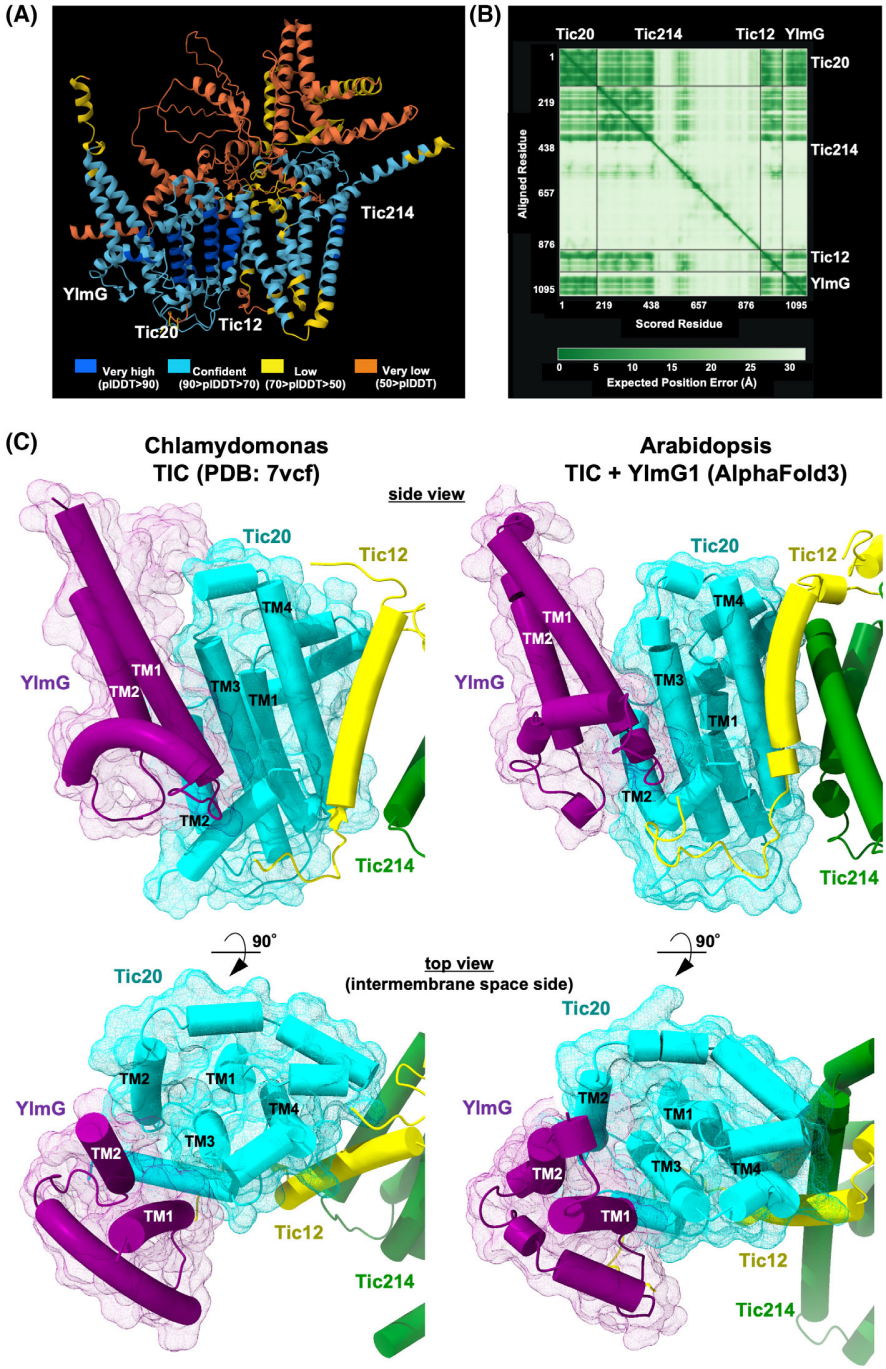
Assessment of structural confidence of the AlphaFold3‐predicted *Arabidopsis* TIC and bound YlmG. (A) The predicted local distance difference test (plDDT) score used by the AlphaFold 3 prediction was depicted with different colors on the predicted 3D model of *Arabidopsis* TIC containing Tic12, Tic20, and Tic214 with bound YlmG. Dark and light blue indicate predicted structures with very high and high confidence, respectively. (B) The predicted alignment error (PAE) heatmap from the AlphaFold 3 prediction. Low PAE score (dark green) indicates that the predicted structural arrangement between the two positions (amino acid residues) running along the axes is of high confidence. (C) Close‐up views of Tic20 and YlmG interaction in the CryoEM structure of *Chlamydomonas* TIC (PDB: 7vcf, left) and in the predicted structure of *Arabidopsis* TIC (right). The predicted model and the various associated scores were retrieved from the AlphaFold server (https://alphafoldserver.com/) powered by AlphaFold3 [21].

For the validation of the predicted structure, Fig. 5A shows the values of the predicted local distance difference test (plDDT), which represent a per‐residue confidence score ranging from 0 to 100 used by the AlphaFold 3 prediction. Especially, the membrane‐embedded part of the complex consisting of Tic20, Tic12, and YlmG exhibited substantially high plDDT scores. The overall interface predicted template modeling score (ipTM) and the predicted template modeling score (pTM) were 0.7 and 0.51, respectively, both of which fell within the range of relatively high confidence. Fig. 5B shows the predicted alignment error (PAE) heatmap from the AlphaFold 3 prediction to assess the confidence of relative positioning between residue pairs. PAE scores between Tic20 and YlmG appeared remarkably low, indicating that the predicted structural arrangement between Tic20 and YlmG was of high confidence. Fig. 5C shows a close‐up view of the Tic20 and YlmG interaction in the CryoEM structure of *Chlamydomonas* TIC (left) and in the predicted structure of *Arabidopsis* TIC (right). In both cases, transmembrane helix 1 (TM1) and TM2 of YlmG position in close proximity to the TM3 of Tic20 in the membrane, while there seems to be no obvious channel‐ or pore‐like structure between the two proteins. Our biochemical data using *Arabidopsis* shown in this study may support an idea that YlmG1 is recruited to this predicted position only when preprotein is being translocated through TIC.

Since YlmG1 is a two‐transmembrane helices‐containing protein, this reminds us to consider structural as well as functional similarities between YlmG1 in chloroplast protein import involving Tic20 and Mgr2 in mitochondrial protein import involving Tim17/Tim23 [22]. Mgr2 is a mitochondrial inner membrane protein carrying two‐transmembrane helices, whereas both Tim17 and Tim23 bear four transmembrane helices. Tim17 and Tim23 have separate, lipid‐exposed cavities that face in opposite directions, of which only Tim17 cavity forms the precursor protein translocation path [23, 24]. During translocation of substrate precursor proteins, Mgr2 likely seals the lateral opening of the Tim17 cavity to facilitate the translocation process and/or to help the lateral release of the translocating protein to the lipid phase and thus functions as a lateral gatekeeper. By analogy, YlmG1 may function as such a lateral gatekeeper at the concave cavity formed by Tic20/Tic12/Tic214 for the incoming preproteins to sort preproteins to be completely translocated into the stroma or those to be released to the inner envelope membrane (Fig. 4, right). While there is no obvious sequence similarity between YlmG1 and Mgr2, these two proteins of distinct evolutionary origins may play functionally equivalent roles in chloroplast TIC and in mitochondrial TIM, respectively.

For the case of TIC involved in chloroplast protein import, such gate‐keeping function may be critically important or absolutely required because that many incoming thylakoid membrane proteins carrying various hydrophobic transmembrane segments should be sorted to be completely translocated but not to be released laterally to the inner envelope membrane. Absolute essentiality of YlmG1 in chloroplast biogenesis may be related to such a gate‐keeping function of YlmG1 associated with TIC. To prove such hypotheses, more biochemical analyses, such as extensive crosslinking experiments between various translocating preproteins and YlmG1 and other TIC constituents will be required in the future. This will need a strong enough highly specific antibody against YlmG1. In addition, high resolution CryoEM structures of TIC with bound translocating preproteins of distinct intraorganellar destinations await being solved in order to elucidate the detailed molecular mechanism of preprotein translocation through TIC.

## Conflict of interest

The authors declare no conflict of interest.

## Author contributions

ML performed most experiments and wrote the manuscript. XZ performed experiments in the initial stage of this study. MN conceived and designed the research, analyzed the data, and wrote and revised the manuscript. All authors read and approved the final version of the manuscript.

## Data Availability

All data supporting the findings of this study are available in the article.
